# Improving continuity of care through an electronic messaging system

**Published:** 2012-09-04

**Authors:** Line Melby, Merete Lyngstad, Ragnhild Hellesø

**Affiliations:** Researcher, Institute of Health and Society, University of Oslo, Oslo, Norway; Researcher, Institute of Health and Society, University of Oslo, Oslo, Norway; University of Oslo, Institute of Health and Society, Norway

**Keywords:** home care, collaboration, information, electronic message, information and communication technology

## Abstract

**Background:**

An increasing number of patients will receive municipal home health care services. Most of these patients will have frequent contact with a number of care providers across different settings, including GPs and hospitals. Ineffective transfer of information between the providers threatens the patient safety and quality of care.

**Purpose:**

The purpose of this Norwegian ongoing study, ‘Bridging the Information Gap in Patient Transition’, is to investigate how the introduction of an electronic messaging (e-message) system affects the quality of collaboration between home care providers and GPs.

**Theory:**

We draw on Haggerty et al.'s (2003) conceptualization of informational continuity of care.

**Methods:**

We use a combination of semi-structured interviews with care providers and participant observation of home care nursing.

**Results and conclusions:**

Our study shows that the e-message system may contribute to improved communication between home care and GPs, compared to ‘traditional’ communication (telephone, face to face meetings). Although still used on a small scale, the introduction of e-messaging points towards more accurate and less obtrusive communication, and the communication is simultaneously documented in the patient’s medical record. Two areas that we see may have profound consequences for patient care are highlighted: the medication process and the accuracy of information.

